# The Effectiveness of Hepatic Arterial Infusion Chemotherapy with 5-Fluorouracil/Cisplatin and Systemic Chemotherapy with Ramucirumab in Alpha-Fetoprotein-Producing Gastric Cancer with Multiple Liver Metastases

**DOI:** 10.1155/2018/5402313

**Published:** 2018-11-11

**Authors:** Yasuhiro Doi, Yasushi Takii, Kenji Mitsugi, Koichi Kimura, Yutarou Mihara

**Affiliations:** ^1^Department of Oncology, Hamanomachi Hospital, Fukuoka, Japan; ^2^Department of Internal Medicine, Munakata Medical Association Hospital, Munakata, Japan; ^3^Department of Surgery, Munakata Medical Association Hospital, Munakata, Japan; ^4^Department of Pathology, Kurume University Hospital, Kurume, Japan

## Abstract

Alpha-fetoprotein- (AFP-) producing gastric cancer (AFPGC) is characterized by a high incidence of liver and lymph node metastases and poor prognosis. Although several case reports have described successful multidisciplinary treatment, there are currently no standard therapies for AFPGC. A 57-year-old man presented with upper abdominal pain. His serum AFP level was extremely high (588.9 ng/mL). Computed tomography (CT) revealed multiple liver metastases with several lesions at an imminent risk of rupture. Five days after admission to our hospital, one lesion ruptured. Transarterial chemoembolization (TACE) of the ruptured tumor was performed, and hepatic arterial infusion chemotherapy (HAIC) with 5-fluorouracil (5-FU)/cisplatin (CDDP) to the other liver metastases was administered. The patient's AFP levels decreased to 297.1 ng/mL. Gastrointestinal endoscopy revealed Borrmann type 2 lesion in the pyloric portion. Pathological examination indicated hepatoid adenocarcinoma of the stomach and metastatic liver. The final diagnosis was AFPGC and multiple liver metastases. The patient underwent systemic chemotherapy with capecitabine/CDDP (cape/CDDP) for three months. His AFP level increased extremely, and CT revealed progression of the liver metastases. TACE was performed, and HAIC (5FU/CDDP) was administered to the progressive lesion of the liver. Originating from the gastric lesion, a distal gastrectomy and D2 + *α* lymph node resection were performed. One month after the operation, the patient underwent systemic chemotherapy with paclitaxel/ramucirumab (PTX/RAM). After eight cycles of chemotherapy, his AFP level had declined, and CT showed a complete response. After three months of drug withdrawal, the patient has undergone maintenance treatment with RAM. It has been two years since the recurrence. Our experience suggests that HAIC with 5-FU/CDDP and systemic chemotherapy with a regimen including RAM may be an effective treatment for AFPGC.

## 1. Introduction

Alpha-fetoprotein- (AFP-) producing gastric cancer (AFPGC) is a relatively rare type of malignancy that comprises about 1.1–8.8% of all gastric cancers [1–3]. AFPGC is characterized by a high incidence in the liver and lymph nodes and poor prognosis [1–3]. There is no standard therapy for this subtype of gastric cancer. However, several case reports have described successful multidisciplinary therapy.

In this study, we presented a case of AFPGC in which the patient has remained alive for 30 months without recurrence after receiving distal gastrectomy with D2 + *α* dissection, hepatic arterial infusion (HAI), and systemic chemotherapy with paclitaxel/ramucirumab (PTX/RAM).

## 2. Case Report

**Figure 1:**
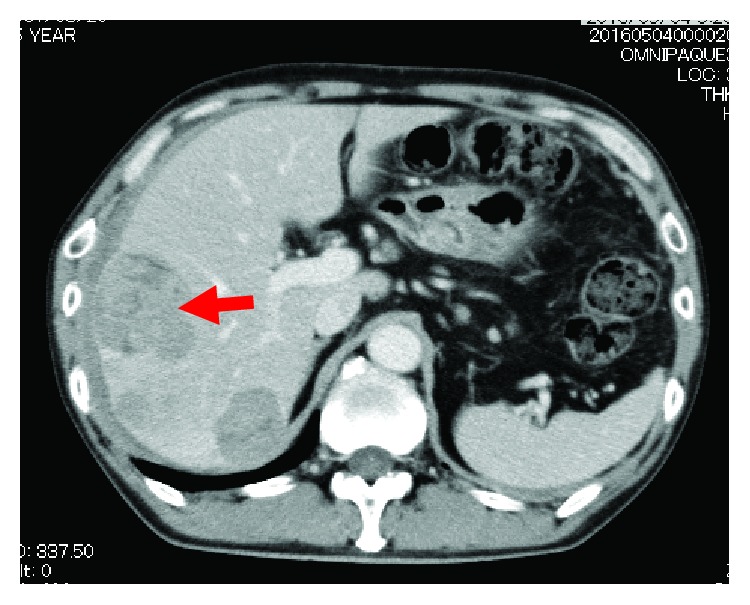
CT scan showing multiple liver metastases suggestive of hepatocellular carcinoma. The red arrow indicates the mass rupturing imminently.

**Figure 2:**
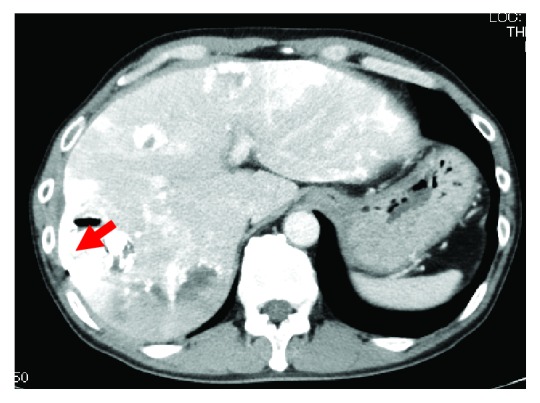
CT scan showing the liver after TACE and HAIC. The red arrow indicates the site of the ruptured mass.

**Figure 3:**
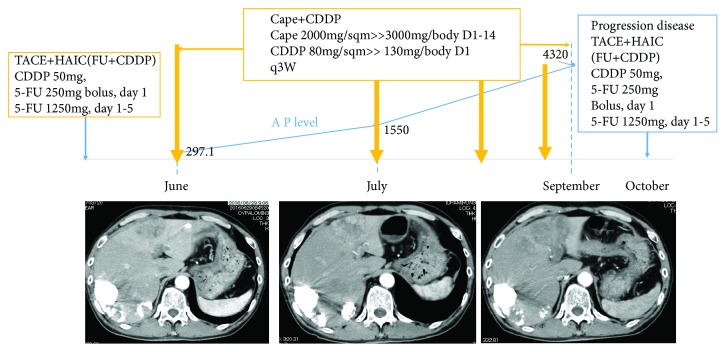
Timeline of patient course and treatment. The blue line indicates the AFP level. The patient received four cycles of chemotherapy with cape/CDDP. His AFP level increased from 297.1 to 4320 ng/mL. CT scans revealed the growth of liver metastases.

A 57-year-old man presented to the internal medicine department with complaints of sudden upper abdominal pain. He denied fever, changes in the color of his urine or stool, nausea, or vomiting. Physical exams revealed no remarkable findings. Laboratory examination showed a white blood cell (WBC) count of 10,600/*μ*L, hemoglobin (Hb) of 14.6 g/dL, C-reactive protein (CRP) of 2.49 mg/dL, blood urea nitrogen (BUN) of 9.5 mg/dL, serum creatinine (Crea) of 0.63 mg/dL, aspartate aminotransferase concentration of 25 U/L, alanine aminotransferase concentration of 19 U/L, lactate dehydrogenase concentration of 241 U/L, alkaline phosphatase (ALP) concentration of 338 U/L, *γ*-glutamyl transpeptidase (*γ*-GTP) concentration of 66 U/L, serum total protein concentration of 6.65 g/dL, and serum albumin concentration of 3.59 g/dL. His serum level of AFP was elevated to 588.9 ng/mL, whereas his carbohydrate antigen 19-9 (CA19-9) and carcinoembryonic antigen (CEA) levels were within the normal ranges. An abdominal computed tomography (CT) scan revealed multiple tumors on his liver suggestive of hepatocellular cancer with part of the tumor rupturing imminently (Figure 1). Five days after admission to our hospital, the mass of the liver ruptured. He was transferred to a different hospital and underwent transarterial chemoembolization (TACE) of the rupturing lesion and HAIC with 5-fluorouracil (5FU)/cisplatin (CDDP) to the others. The patient's AFP levels decreased from 588.9 to 291.7 ng/mL after one cycle of HAIC with 5FU/CDDP (Figures 2 and 3).

**Figure 4:**
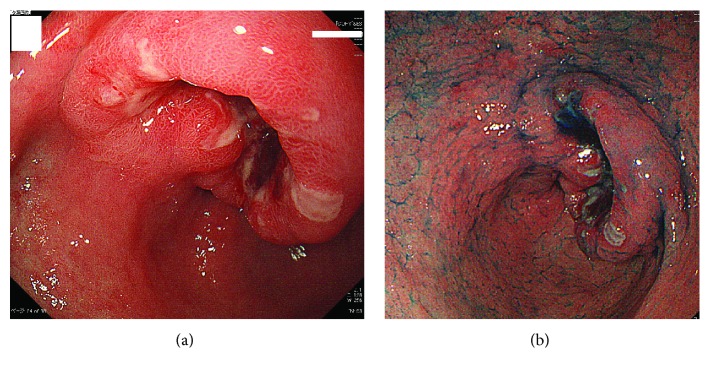
Gastrointestinal scope showing the Borrmann type 2 lesion on the pyloric portion of the lesser curvature that was histologically diagnosed as suspected hepatoid adenocarcinoma.

A gastrointestinal scope after TACE and HAIC showed a Borrmann type 2 lesion on the pyloric portion of the lesser curvature, which was histologically diagnosed as suspected hepatoid adenocarcinoma (Figure 4).

**Figure 5:**
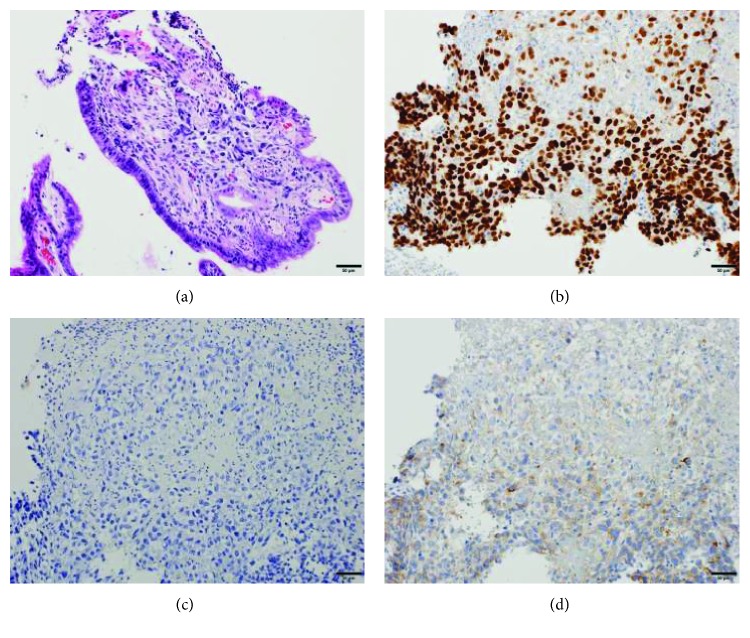
Gastric biopsy. (a) Hematoxylin and eosin staining indicating suspected hepatoid adenocarcinoma. (b) Positive staining for SALL4. (c) Negative staining for AFP. (d) Focally positive staining for glypican3.

**Figure 6:**
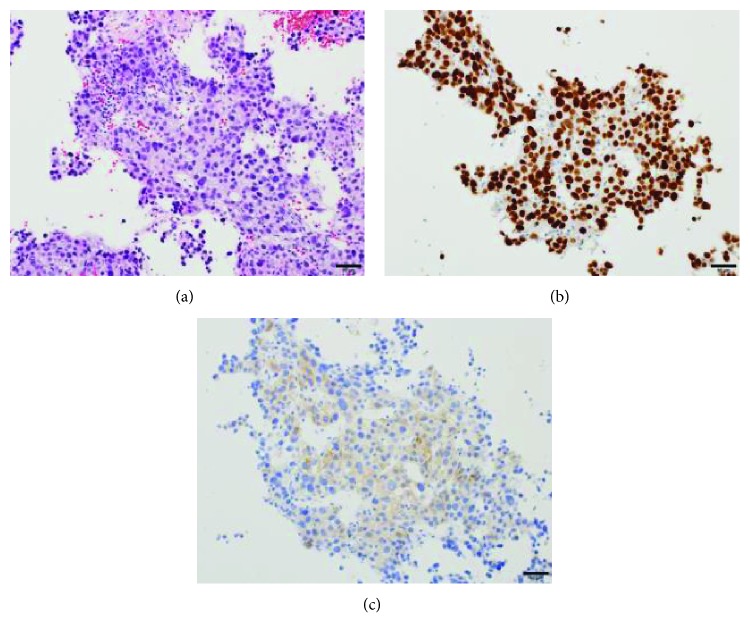
Hepatic biopsy. (a) Hematoxylin and eosin staining indicating that the lesion is a liver metastasis. (b) Positive staining for SALL4. (c) Focally positive staining for heterochromatin protein 1 (HP1).

Immunohistological staining for AFP and Sal-like protein 4 (SALL4), glypican3, and human epidermal growth receptor 2 (HER2) was negative for AFP and positive for SALL4 and Glypican3 (Figure 5). A liver biopsy confirmed that the lesion was a liver metastasis. Analysis of the liver biopsy specimen revealed that the liver mass was metastatic carcinoma of the liver. Immunohistological staining showed that the specimen was positive for SALL4 and heterochromatin protein 1 (HP1) (Figure 6). Based on these results, the patient was diagnosed with AFPGC and multiple liver metastasis.

**Figure 7:**
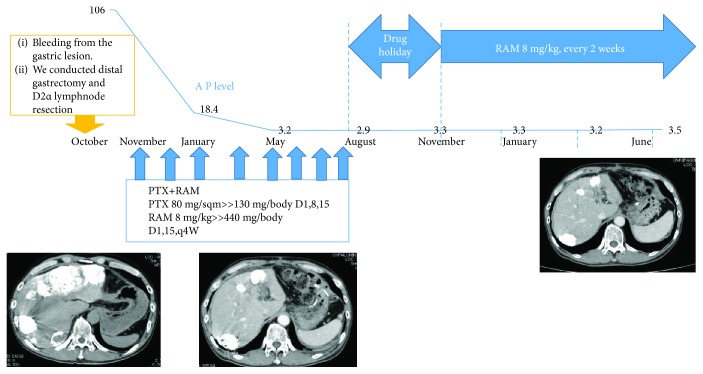
After surgery, the patient received eight cycles of chemotherapy comprising PTX and RAM. His AFP levels decreased within the normal range and the lesion in the liver disappeared on a CT scan. After a three-month drug holiday, RAM monotherapy was initiated.

He underwent four cycles of systemic chemotherapy with capecitabine (cape)/cisplatin (CDDP) (cape 2000 mg/sqm >> 3,000 mg/body days 1–14, CDDP 80 mg/sqm >> 130 mg/body day 1, every 3 weeks), which resulted in progressive disease. His AFP levels increased from 297.1 to 4320 ng/mL, and a CT scan revealed progression of the liver metastasis (Figure 3). As the lesion of the liver on S4 was at a high risk of rupturing and HAIC was effective in the previous treatment, we decided to perform TACE and HAIC to the liver metastasis again. Bleeding developed from the gastric lesion after a cycle of HAIC with 5-FU/CDDP, and the patient underwent a distal gastrectomy and D2 + *α* lymph node resection. One month after the gastrectomy, eight cycles of systemic chemotherapy comprising PTX and RAM were administered. As a result, the patient's AFP levels decreased to 2.9 ng/mL and a CT scan showed that the tumor had vanished (Figure 7). After a three-month drug holiday, chemotherapy with RAM as a maintenance therapy was resumed (Figure 7). The patient has continued with RAM and has so far survived for 19 months after recurrence and is alive without a recurrence.

## 3. Discussion

AFPGC comprises 1.1–8.8% of all gastric cancers and is a highly aggressive type of gastric cancer compared to non-AFPGC [1–3]. Liver metastasis is frequently observed simultaneously, and metachrony occurs because of the biological characteristics that tend to cause vascular invasion [4–6].

AFPGC has been classified into four histological subtypes: hepatoid, yolk sac tumor, enteroblastic, and common adenocarcinoma type. Positive AFP staining is observed in 54% and 30% of hepatoid and yolk sac tumors, respectively. In recent reports, glypican3 and SALL4 staining in addition to AFP protein measurement are effective for the diagnosis of AFPGC. Ushiku and Fukayama reported that, while the AFP-positive staining rate was low (16% in 32 cases of gastric cancer), the rates for glypican3 and SALL4 were 56% and 78%, respectively [7]. Although AFP is negative by staining, our case was diagnosed as AFPGC due to findings suggestive of hepatoid adenocarcinoma, SALL4- and glypican3-positive staining, and high serum AFP level.

HAIC is considered effective for the treatment of the liver metastasis of AFPGC. Local intra-arterial chemotherapy such as HAIC has a lower frequency of side effects than that of systemic chemotherapy. Additionally, HAIC is effective because high concentrations of the drug can be injected locally. Sato et al. reported that the combination of HAIC with 5-FU/epirubicin and systemic chemotherapy with S-1 for his patient with AFPGC was effective and safe [8]. There are 17 reports in Japan from 1996 to 2016. Among them were three cases in which HAIC with 5-FU/CDDP was performed. The patients experienced partial response (PR) or complete remission (CR) and have obtained long-term survivals of two years or more after relapse [9–11] (Table 1).

This case was diagnosed as AFPGC with synchronous multiple liver metastases. For liver metastasis rupture during initial treatment, TACE and HAIC with 5FU/CDDP were performed for volume control. Systemic chemotherapy with cape/CDDP was administered for four cycles. The disease was less responsive to cape/CDDP systemic chemotherapy and developed to progressive disease (PD). As HAIC with 5-FU/CDDP and TACE was initially effective for the liver metastasis in this case, it was repeated. HAIC and TACE effectively controlled the liver metastasis in this case.

AFPGC is very rich in angiogenesis compared to other gastric cancers [12]. Most cancer cells produce vascular endothelial growth factor (VEGF), which is involved in the mitosis of endothelial cells in vitro and is a factor of angiogenesis in vivo [13, 14]. VEGF-C is an isoform of VEGF that acts as a factor in lymphangiogenesis and promotes lymph ductogenesis. According to Kamei et al., VEGF is highly expressed in AFP-producing gastric cancer compared to the expression in non-AFP-producing gastric cancer [15]. The vascular-rich nature of the liver and lymph node metastases is likely related to VEGF or VEGF-C. RAM inhibits VEGF-A, VEGF-C, and VEGF-D by blocking VEGF-R2, and the same targeted drug may be effective for AFP-producing gastric cancer. In the present case, RAM was very effective, leading to a CR after eight cycles of PTX/RAM as adjuvant chemotherapy. This case is the second in Japan to report the effectiveness of RAM.

## 4. Conclusion

Our experience suggested that HAIC with 5-FU/CDDP and systemic chemotherapy with a regimen including RAM may be an effective treatment for AFPGC. HAIC may be effective for the local control of liver metastasis, while RAM may be effective for suppressing progression in the whole body.

## Figures and Tables

**Table 1:** In Japan, from 1996 to 2016, there are four cases reported in which HAIC with 5FU/CDDP was performed. All of them are a good prognosis.

Author (year)	Age	Sex	Rejimen of systemic chemotherapy	Other therapies	Effect	Prognosis
Ozaki et al. [9] (2008)	73	Male	—	Gastrectomy + D3 resection Partial hepatectomy	PR	Unknown
Takada et al. [10] (2008)	75	Female	DTX→PTX→CPT-11	Gastrectomy Partial hepatectomy RFA, TAE, radiation	CR	42 months progression free
Tanizaki et al. [11] (2009)	65	Female	S-1	Distal gastrectomy	CR	28 months progression free
Our case (2016)	57	Male	Cape/CDDP→PTX/ramucirumab→ramucirumab	TACE Distal gastrectomy + D2*α* resection	CR	30 months progression free
